# Expression genetics identifies spinal mechanisms supporting formalin late phase behaviors

**DOI:** 10.1186/1744-8069-6-11

**Published:** 2010-02-11

**Authors:** Xiangqi Li, Peyman Sahbaie, Ming Zheng, Jennifer Ritchie, Gary Peltz, Jeffrey S Mogil, J David Clark

**Affiliations:** 1 Veterans Affairs Palo Alto Healthcare System, Palo Alto, CA, USA; 2 Department of Anesthesia, Stanford University School of Medicine, Stanford, CA, USA; 3 Department of Psychology and Alan Edwards Centre for Research on Pain, McGill University, Montreal, QC, Canada

## Abstract

**Background:**

Formalin injection into rodent hind paws is one of the most commonly employed pain assays. The resulting nocifensive behaviors can be divided into two phases differing in timing, duration and underlying mechanisms. Spinal sensitization has long been felt to participate in the second phase of this response, although this sensitization is incompletely understood. By using correlative analysis between spinal gene expression and mouse strain-dependent intensity of late phase behavior, we hypothesized genes participating in variability of the response could be identified.

**Results:**

Late phase formalin behavior scores among 10 inbred mouse strains were correlated with a spinal cord gene expression database constructed using expression arrays. Messenger RNA levels for several genes were highly correlated with the late phase behavioral responses. Most of these genes had already been implicated in mechanisms regulating pain and analgesia. One of the most strongly correlated genes, *Mapk8 *coding for c-Jun N-terminal kinase 1 (JNK1), was chosen for further analysis. Studies using additional strains of mice confirmed that spinal cord mRNA expression levels of *Mapk8 *followed the pattern predicted by strain-specific levels of formalin behavior. Interestingly, spinal cord JNK1 protein levels displayed an inverse relationship with mRNA measurements. Finally, intrathecal injections of the selective JNK inhibitor, SP600125, selectively reduced late phase licking behavior.

**Conclusions:**

Wide differences in pain behaviors, including those resulting from the injection of formalin, can be observed in inbred strains of mice suggesting strong genetic influences. Correlating levels of gene expression in tissues established to be mechanistically implicated in the expression of specific behaviors can identify genes involved in the behaviors of interest. Comparing formalin late phase behavior levels with spinal cord gene expression yielded several plausible gene candidates, including the *Mapk8 *gene. Additional molecular and pharmacologic evidence confirmed a functional role for this gene in supporting formalin late phase responses.

## Background

The injection of formalin into the skin of rodent hind paws to cause spontaneous pain-related (nocifensive) behaviors is one of the most commonly used animal pain assays [1]. This test was introduced in 1977 as a method that allowed nocifensive behaviors to be studied without restraint, and with a continuous rather than transient source of stimulation [2]. This model can be distinguished from many other irritant pain models--for example, ones involving the administration of carrageenan, bee venom, capsaicin and other compounds--by the existence of a biphasic response. An intense first (early) phase of hindpaw shaking and licking subsides approximately 5-10 minutes after formalin injection, only to have the behaviors reappear and last another 30 minutes or longer. The first phase of this test is thought to be due to direct effects of formalin on nociceptive fibers [3], and recent evidence suggests that the Transient receptor potential cation channel 1 (TRPA1) receptor/ion channel might mediate that signal transduction; TRPA1-deficient mice and mice administered a selective TRPA1 antagonist display greatly reduced early phase formalin-induced behaviors [4]. Formalin early phase behavior are sensitive to reversal by analgesics such as opioids and paracetamol [3,5].

The second (late) phase of the formalin response, sometimes referred to as the "inflammatory phase," has classically been ascribed to inflammation as non-steroidal anti-inflammatory drugs such as acetylsalicylic acid, ibuprofen and ketoprofen are active in reducing the associated behaviors [5,6]. However, many drugs without anti-inflammatory activity are also active in this phase including gabapentin, lamotrigine, nitric oxide synthase (NOS) inhibitors and others [7,8]. Further exploration of the basis for late phase nocifensive behaviors has revealed that sensitization of dorsal horn neurons is involved [9,10]. In fact, the intrathecal injection of many agents reduces late phase behaviors. Late phase behavior is also of interest because of the similarities in presumed mechanism between it and some dimensions of neuropathic pain [11].

Large inter-individual differences exist between both humans and animals with respect to pain, nociceptive sensitivity and analgesic responses [12]. That genetics mediates a significant percentage of inter-strain variance in commonly used mouse pain assays has been firmly established. The formalin test when applied to inbred mice leads to highly strain-dependent results for both early and late phase behaviors [13,14]. Such inter-strain differences have been exploited using quantitative trait locus (QTL) mapping, haplotypic analysis and other techniques to gain insight into the identity of the trait-relevant genes. For example, one recent report used both QTL and haplotypic analyses to demonstrate that the early phase of the formalin response was dependent on the activity of afferent neuron ATPase activity, presumably related to the ability of the neuron to maintain an electrochemical gradient supporting neuronal firing [15]. The heuristic value of the approach was illustrated by the fact that the relevant gene was *Atp1b3 *[15], encoding a little-studied β subunit of the ATPase rather than the catalytic α subunit. Genetics based studies have not yet been published demonstrating how particular genes might be responsible for the wide differences between strains in their late phase behavior despite the importance of this response.

An alternative technique for identifying genes potentially involved in regulating a phenotype of interest is to correlate expression of the phenotype, such as a pain-related nocifensive behavior, with the expression level of genes in a tissue known to take part in controlling the phenotype [16,17]. The assumptions underlying this approach are that the abundance of mRNA for at least some controlling genes will be either positively or negatively correlated with the phenotype of interest, and that the correlation reflects a functional role for the gene in the phenotype. This "expression QTL" or "genetical genomics" technique is particularly attractive as any number of phenotypes could be compared with expression databases for specific tissues. Here we use such an approach to identify candidate genes controlling late phase formalin induced nocifensive behavior in inbred strains of mice.

## Results

### Inter-strain differences in formalin-induced nocifensive behaviors

**Figure 1:**
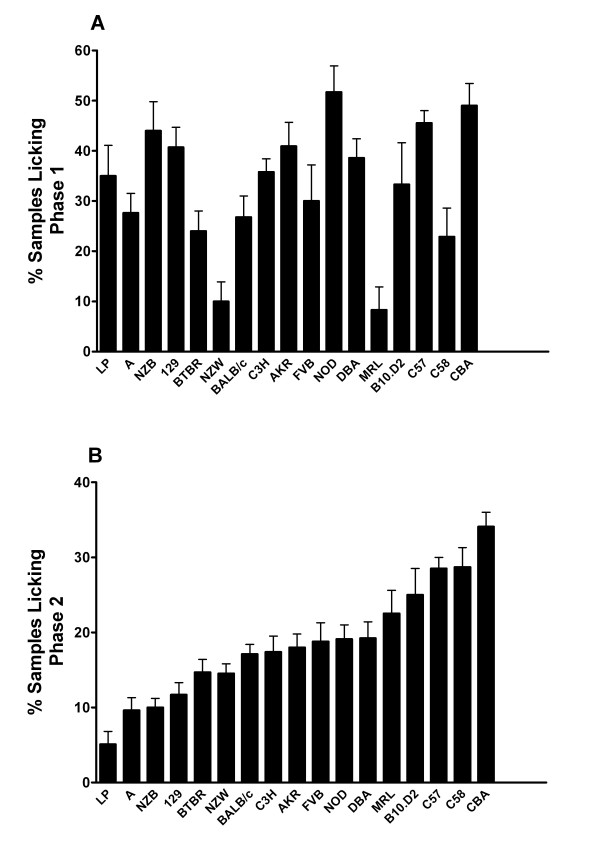
**Formalin-induced nocifensive behaviors**. Video recordings of mice licking/biting after plantar hind paw injection of formalin were reviewed for 17 strains of inbred mice (n = 10-25/strain). Bars represent the mean ± S.E.M. percentage of samples featuring licking/biting in early (0-5 min; graph A) and late phase (10-60 min; graph B).

Early and late phase responses to subcutaneous hind paw formalin injection were determined for 17 strains of inbred mice. The results demonstrated large differences between strains for both phases during the period of observation (Figure 1). The strain specific licking/biting times were converted to z-scores for the purpose of performing subsequent correlative analysis, and those scores appear in Table 1. Correlational analysis of the rank order of responses for the two phases revealed no significant statistical correlation (Spearman r = 0.22, p > 0.05).

**Table 1:** Z-Scores for mouse strain specific formalin induced licking behavior.

Strain	Phase I z-score	Phase II z-score
**129**	0.17	-0.64
**A**	-0.39	-0.81
**AKR**	0.18	-0.11
**B10.D2**	-0.14	0.47
**BALB/c**	-0.42	-0.19
**BTBR**	-0.36	-0.33
**C3H**	-0.03	-0.16
**C57**	0.38	0.76
**C58**	-0.59	0.77
**CBA**	0.53	1.22
**DBA**	0.08	-0.01
**FVB**	-0.24	0.06
**LP**	-0.07	-1.18
**MRL**	-1.22	0.26
**NOD**	0.71	0.09
**NZB**	0.32	-0.78
**NZW**	-1.15	-0.40

These values were calculated based on the percent licking data for early and late phase presented in Figure 1.

### Late phase formalin response intensity versus spinal cord gene expression

In preparatory experiments we constructed a spinal cord gene expression database using material from 10 of the 17 strains of inbred mice tested behaviorally. In order to enrich our correlational analysis in associations with strongly differentially expressed genes, and to minimize false-positive associations, probesets were first selected showing 3-fold or greater differential expression across those 10 strains. Thus, data from the >45,000 probesets represented on the array chips was first refined to a group of 2,500 probesets.

Our primary interest in these studies was to identify genes associated with the intensity of formalin late phase behavior. Pearson correlational analysis between late phase behavior and the refined group of 2,500 probesets identified several strongly associated genes. The 5 strongest associations are displayed in Table 2. For 3 of the 5 associations the direction of correlation was negative (lower expression with higher levels of late phase behavior). All 4 known genes identified, or isoforms of the identified genes, had been previously associated with pain or analgesia-related behaviors in rodent pain models (see references in Table 2). For 2 of the genes, coding for cytosolic phospholipase A2 and the SCN2B sodium ion channel subunit, a role for their protein in controlling late phase formalin behavior had been previously reported. Because the *Mapk8 *gene, coding for c-Jun N-terminal kinase 1 (JNK1), was also strongly associated with spinal pain mechanisms but has been less strongly investigated with respect to controlling formalin late phase behaviors specifically, we chose to pursue this association further.

**Table 2:** Genes associated with late phase formalin licking.

Gene	Protein	Correlation	Pain/Analgesia Models	**References**^†^
*Pla2g4e*	Phospholipase A2 (PLA2)	-0.96	Formalin Nerve injury Carrageenan	[27,28,39,40] [40] [28,40]
*Mapk8*	c-Jun N-terminal kinase 1 (MAPK8, JNK1)	-0.91	Nerve Injury	[20]
RIKEN 4833416E15	(Unknown)	0.90	-	
*Scn2b*	Sodium channel beta2 subunit	0.89	Formalin	[30]
*Kcnj9*	G-protein-gated inwardly rectifying K^+ ^channel subunit 3 (Kir3.3, GIRK3)	-0.89	Morphine	[41]

The results of correlation analysis between strain-specific late phase formalin scores and a spinal cord gene expression database. The top 5 most-strongly-linked genes and the proteins they encode, along with the correlation coefficients and the direction of correlation is provided. Positive correlation signifies greater gene expression corresponding to greater levels of pain behavior.

^†^References for pain-related investigations specific to the named gene or closely related gene family members.

### The polymorphic nature of the mitogen activated protein kinase 8 (*Mapk8*) gene

**Figure 2:**
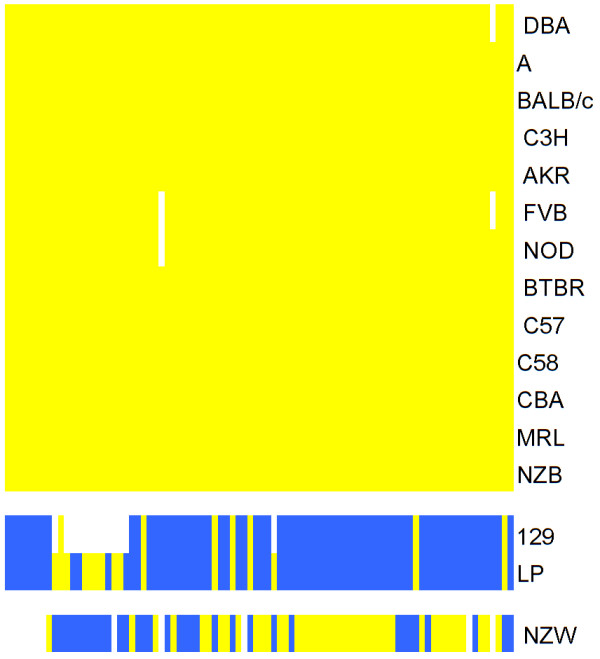
**Haplotypic structure of the *Mapk8 *gene**. Using SNP data for the 13 inbred mouse strains, for which it was available, the strains partitioned into 3 haplotypes. There were 86 SNPs available for this analysis. In this figure blue represents the common allele, yellow represents the minor allele, and white represents missing alleles.

Using publicly available data, all SNPs within 2 kb of the *Mapk8 *gene were identified, totaling 86 SNPs [18]. The data selected were restricted to the 13 inbred strains of mice present in that database represented in our experiments. Three distinct haplotypes emerged from this analysis with 10 strains sharing the most common configuration of alleles (Figure 2). All three strains having unique haplotypes (129/SvIm, LP and NZW/LaC) displayed late phase responses near the lower end of the response spectrum.

Haplotypic analysis using the method of Wang et al. was conducted to determine the degree of association of the *Mapk8 *haplotype with the strain specific data [19]. The results showed weak but significant statistical correlation (*p *= 0.029) with *Mapk8 *haplotype potentially accounting for 54.4% of the variance in the late phase data.

### Additional mRNA and protein analysis of *Mapk8 *in spinal cord tissue

**Figure 3:**
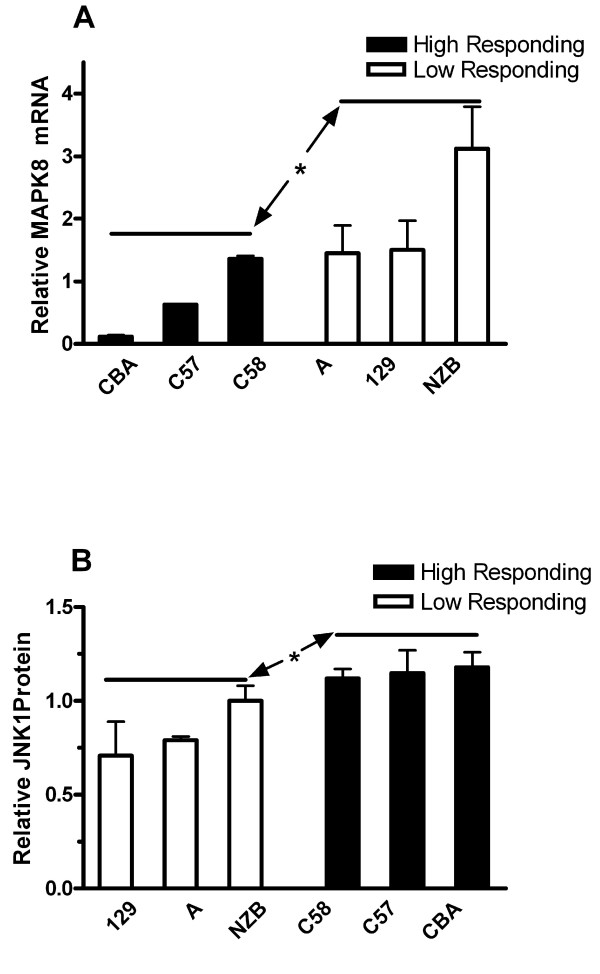
**Analysis of spinal cord *Mapk8 *expression in supplemental strains**. In these experiments both real time qPCR and Western analysis were used to quantify mRNA and protein levels respectively. Graph A contains the results of qPCR experiments in which the relative levels of *Mapk8 *mRNA for high late phase responding strains were compared with low responding ones. In graph B, similar experiments were performed using spinal cord protein homogenates. For the protein experiments the spinal cord levels of JNK1 were normalized to the spinal cord levels in the intermediate-responding BALB/c mice arbitrarily set at a level of 1.0. For all experiments samples from n = 4 mice per strain were analyzed in triplicate. Bars represent mean ± S.E.M. Three strains within each group were compared to other strain group, *p < 0.05.

In order to confirm the association of *Mapk8 *with inter-strain variability in late phase formalin behavior, analyses of the spinal cord levels of mRNA and protein were undertaken. To complete these studies, 4 strains of mice were chosen for which behavioral data were available but for which expression array data was not collected: CBA and C58 (both high formalin late phase responding), and NZB/Bln and A (both low responding). In addition, we included the C57BL/6 and 129SvIm strains for which array data were available. For each of these strains, spinal cord tissue from adult animals was harvested and subjected to real-time qPCR. The results are displayed in Figure 3. The low responding strains correctly partitioned together as more highly expressing *Mapk8 *mRNA, and there was a statistically significant difference in mRNA levels between the high and low responding strains.

We went on to complete Western blot experiments to quantify spinal cord levels of the corresponding JNK1 protein using the same set of strains plus the intermediate-responding BALB/c strain that was used to normalize the measured protein levels. Figure 3 shows again that high- and low-responding strains partitioned together with respect to spinal cord protein levels. Importantly, mRNA and protein levels were *inversely *correlated: strains with low mRNA levels had higher protein levels. The BALB/c strain had a protein expression level intermediate to the high and low responding groups (data not shown). The differences in spinal cord protein levels of JNK1 between the high and low responding strains were also statistically significant.

### Pharmacological inhibition of JNK1 selectively reduces late phase formalin behavior

**Figure 4:**
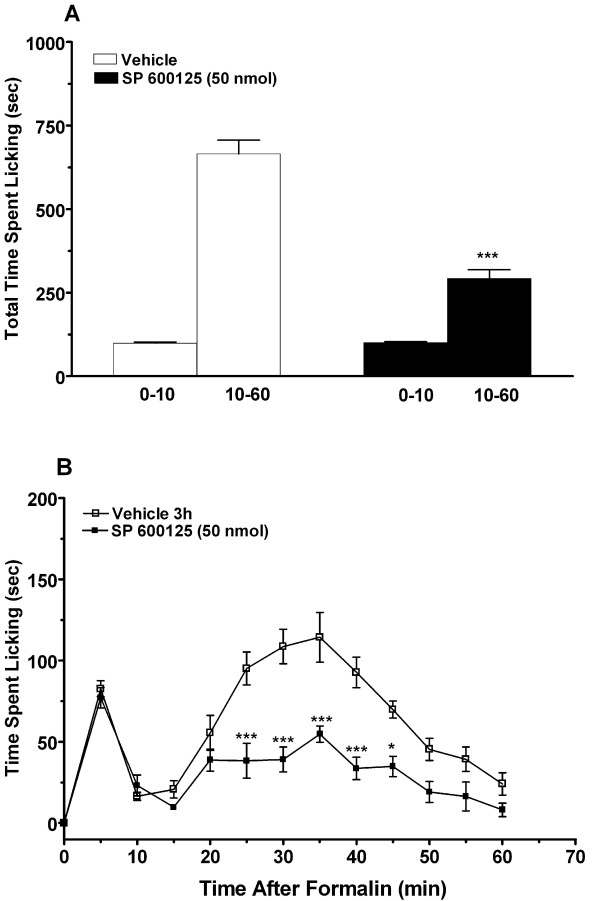
**The effects of the JNK inhibitor, SP 600125, on formalin-induced nocifensive behaviors**. For these experiments groups of mice (n = 8) first underwent intrathecal injection of SP 600125 or vehicle 3 h prior to formalin injection. The total duration of licking/biting time during early (0-5 min) and late phase (10 k-60 min) of the formalin response were measured and averaged for either the entire phase (graph A) or in 5-min intervals (graph B). Bars and symbols represent mean ± S.E.M. *p < 0.05, ***p < 0.001 compared to the vehicle group.

The selective JNK inhibitor, SP600125, was used to test the functional link between *Mapk8 *and late phase formalin behavior. The drug was injected intrathecally to target spinal tissue as the relevant source of JNK1 activity. Figure 4 displays results demonstrating that 50 nmol SP600125 significantly and selectively reduced late phase behavior. A higher dose of inhibitor was employed in additional experiments (150 nmol); no early phase and no additional late phase inhibition was observed (data not shown).

## Discussion

The goal of the present studies was to use the emerging technique of correlating pain-related (nocifensive) behavior across multiple strains of inbred mice with genome-wide gene expression in a tissue known to regulate that behavior (i.e., an eQTL or genetical genomics approach). In this case, the late phase of formalin-induced nocifensive behavior was correlated with spinal cord gene expression for 10 strains of mice. Subsequently, 4 additional strains were evaluated in experiments designed to test the correlational results. The results of our correlational studies identified several genes with strongly associated spinal expression patterns. Remarkably, 4 out of the top 5 most-strongly associated genes, or closely related members of their gene families, had previously been demonstrated to have links to pain and analgesia. Subsequent use of mRNA and protein analysis in additional strains confirmed the correlational observations for *Mapk8 *(protein known as JNK1), a spinal cord gene with known roles in neuroplasticity. Finally a selective JNK inhibitor blocked late phase formalin behaviors strongly, with no effect whatsoever on early phase behaviors. In retrospect, no early phase effects of this drug could be expected, as there was no statistical correlation between the early phase and late phase behaviors across the strains tested. Thus, working with expression and behavioral databases for a relatively modest number of mouse strains allowed us to identify highly plausible functionally related genes. The total number of inbred strains required for these experiments was low compared to the numbers typically involved in genetic based approaches such as QTL or haplotypic analyses.

The gene chosen for confirmatory experiments, *Mapk8*, has well-established roles in intracellular signaling, including signaling related to nociception. An early report by Yang et al. [20] used microarray analysis on rat spinal cord dorsal horn tissue after peripheral axotomy to identify JNK1 and other members of the JNK family as upregulated after nerve injury, though a functional role for these changes was not demonstrated. Subsequent experiments confirmed that the three splice variants of JNK (JNK1-3) are highly expressed in spinal cord tissue [10]. Using spinal nerve ligation to create a model of neuropathic pain, Zhuang et al. demonstrated that the up-regulation of JNK1 occurred primarily in spinal astrocytes, and that the spinal administration of a selective JNK1 inhibitor blocked the resulting hypersensitivity [21]. Other experiments showed that selective JNK inhibition reduced nociceptive sensitization in a model of diabetic neuropathy [22] and after bee venom or capsaicin injection [23,24]. The suggested mechanism for activation of JNK1 is via transforming growth factor-activated kinase 1 (TAK1), a member of the MAPK kinase family. Spinal knock-down of TAK1 reduces astrocytic JNK1 phosphorylation in spinal cord tissue after nerve injury [25]. Additional evidence suggests that spinal TNFα may work through JNK1 to produce the cytokine MCP-1, ultimately sensitizing spinal dorsal horn neurons [26].

Overall, the JNK family of MAPK genes is perhaps the least well-studied of the MAPK gene superfamily. Hassanzadeh et al. demonstrated that this gene was required for the appearance of spinal cord "dark neurons" after peripheral formalin injection, neurons which are thought to have undergone excitotoxic injury [27]. It does appear that spinal cord dorsal horn astrocytes are activated after peripheral formalin injection [28]; astrocytes may be the primary site of spinal *Mapk8 *expression as noted above. To our knowledge, the role of *Mapk8*/JNK1 in regulating late phase formalin behavior has not been well studied. Our experiments are, therefore, the first to demonstrate directly a role for *Mapk8*/JNK1 in spinal cord sensitization after formalin injection.

One interesting feature of our findings was the inverse correlation of spinal cord *Mapk8 *mRNA and JNK1 protein levels. Thus mouse strains with higher licking times had greater spinal cord JNK1 protein levels as expected, based on the reported relationship between JNK1 function and pain behaviors in other models. In addition, blockade of JNK using a selective agent reduced late phase formalin licking behavior. However, as demonstrated by the negative correlation coefficient for the mRNA-behavior relationship in Table 2 and confirmatory data in Figure 3, the behaviorally higher responding strains had lower *Mapk8 *mRNA levels. These observations suggest that the steady state levels of JNK1 are controlled by factors other than transcription. One possible factor would be the protein's high stability resulting in a low turnover rate, as JNK1 is constitutively expressed in the spinal cord [21]. Therefore, relatively low levels of *Mapk8 *mRNA would be necessary to maintain the steady state levels of the protein. Another possibility would be that the genetic factor(s) explaining the inter-strain formalin behavioral differences might involve control of translation rather than transcription. In this scenario, genetically regulated enhancement of translation may lead to high protein levels with compensatory reduction in transcriptional activity thus lowering mRNA levels. Control of translation is a complex process [29]. Though our studies did not specifically address mechanisms which could explain this phenomenon, micro RNA (miRNA) is an example of a type of regulatory molecule potentially enhancing translation of multiple genes simultaneously. Alternatively, under basal conditions the *Mapk8 *would be under negative regulation by the JNK1 and its substrates, as intense and prolonged JNK activation would result in programmed cell death[30]. Members of the MKKs (MAPK kinases) which activate JNK pathway are themselves under negative regulation by substrates of JNK, whilst MKPs (MAP kinase phosphatase) which inhibit the pathway are positively regulated by some of the same substrates [31,32]. It is notable that our haplotypic analysis was only weakly suggestive of a role for *Mapk8 *genetics in controlling late phase formalin behavior. In fact two of the other associated genes also had strongly negative correlations between mRNA levels and behavior.

While our experiments focused on *Mapk8*, one other gene (*Pla2g4e*) was statistically even more closely linked to formalin test second phase behaviors. This gene codes for a cytosolic form of phospholipase A2 (PLA2), an enzyme family with strong links to inflammatory pain. PLA2 serves to liberate arachidonic acid which is subsequently metabolized by cyclooxygenase 1 and 2 (COX1/COX2) to form pro-nociceptive prostanoids, although arachidonic acid is a substrate in other pathways as well. This enzyme family is comprised of approximately 15 groups and four main types, including the secreted sPLA2, cytosolic cPLA2, calcium-independent iPLA2, and platelet activating factor (PAF) [33]. Though the *Pla2g4e *isoform has not been examined specifically in pain models, cytosolic PLA2 probably does contribute to spinal sensitization in several models of pain. For example, the cytosolic PLA2 inhibitor, AACOCF3, reduced both nociceptive sensitization and spinal arachidonic acid production in the spinal cord tissue of rats used in the chronic constriction injury model of neuropathic pain [34]. In another set of experiments it was demonstrated that antisense knockdown of spinal cytosolic group 4a PLA2 reduced late but not early phase formalin-induced pain behaviors [35]. This form of phospholipase A2 is the predominant one in spinal cord tissue [36]. In the same group of studies it was determined that spinal cord dorsal horn neurons may be the predominant site of spinal expression of this enzyme. While the results from our study associated a distinct member of the same cytosolic type of PLA2, independent confirmation of this association is required. Unfortunately the pharmacological and immunohistochemical tools currently available would not provide definitive evidence for the participation of *Pla2g4e *in late phase formalin behavior, and it is likely that experiments with knockout mice or spinal knock-down would need to be performed.

Another associated gene with pre-existing data linking its function to formalin late phase behaviors is the *Scn2b *gene coding for the sodium ion channel β_2 _subunit. The β subunits of sodium ion channels modulate the kinetics and other electrical properties of sodium ion channels [37]. In a recent report in which *Scn2b *knockout animals and neurons derived from these animals were studied, it was discovered that *Scn2b *deletion both reduced TTX-sensitive sodium ion currents in small diameter neurons, and the expression of specific sodium ion channels, notably Na_V_1.7 [38]. Critically, gene deletion reduced late phase formalin behaviors without altering early phase responses. Interestingly, Na_V_1.7 is an ion channel whose dysfunction in humans has been associated with either pain hypersensitivity or insensitivity depending on the type of genetic alteration present [39].

## Conclusions

In this report we began with the nocifensive behavior data for a relatively modest number of inbred mouse strains, and using an expression dataset from spinal cord tissue we were able to identify several highly plausible genes for controlling late phase formalin test behavior. Note that this strategy, like conventional QTL mapping and haplotypic analysis, differs from other molecular genetic techniques in that it can identify genes contributing to trait *variability *and not just genes that are trait-relevant. The use of confirmatory expression and pharmacological studies identified *Mapk8*/JNK1 as a key regulator of variable second phase formalin test pain behavior. In addition to the value of identifying this specific target for potential analgesic development, we have provided evidence demonstrating the utility of undertaking this type of correlative study to uncover pain-related signaling mechanisms. The same method if applied to other sorts of pain models might involve the use of expression datasets based on alternative tissues (e.g., dorsal root ganglion) or data sets constructed from animal tissues enduring more chronic pain states (e.g., nerve injury models). Unavoidable, however, will be the need for strong confirmatory data regardless of the technique used to establish the preliminary association. Correlative expression analysis appears to be a viable approach in the expanding realm of genetic approaches to understanding pain mechanisms.

## Methods

### Animals

Mice for all experiments were purchased from The Jackson Laboratory (Bar Harbor, ME) at 6-12 weeks of age, or bred in-house from stock mice so obtained. Upon weaning (at 18-21 d) or immediately after arrival, mice were housed in standard shoebox cages of 2-4 with same sex littermates in a temperature-controlled (20° ± 1°C) environment (14 h: 10 h light/dark cycle; lights on at 07:00 h), and with ad lib access to food and water. Purchased mice were habituated to the laboratory for at least one week before any behavioral testing commenced. Husbandry conditions were similar for mice housed at the McGill University and Palo Alto VA Medical Center (VAPAHCS). All experiments were approved by the local Institutional Animal Care and Use Committees of McGill University and VAPAHCS.

### Formalin test

Mice were habituated in individual transparent Plexiglas cylinders prior to a subcutaneous injection of 5% formalin into the plantar right hindpaw (20 μl volume). Following injection mice were digitally videotaped from underneath a glass floor for 60 min, or directly observed. Video files were later sampled for 5 s at 1-min intervals, and the presence or absence of right hindpaw licking/biting nocifensive behaviors in that 5-s period was scored using Observer software (Noldus, Leesburg, VA). Direct observations timed the licking/biting behavior using a stopwatch. The early phase of the formalin test was defined as 0-5 min post-injection, and the late phase as 10-60 min post-injection. These data were tabulated as the percent of samples licking during the specified intervals. In pharmacological experiments mice were continuously observed providing the total number of seconds spent licking/biting in each phase for each animal.

### Drug administration

The non peptide JNK inhibitor, SP600125 (anthra [1,9-cd]pyrazol-6(2H)-one), was purchased from Sigma (St. Louis, MO) and prepared in DMSO (20% final concentration). Intrathecal (*i.t*.) injections of 5 μl of SP600125 or vehicle (20% DMSO) were made under brief isofluorane anesthesia with a 30-gauge needle between the L5 and L6 level as we have described previously[17]. The SP600125 dose of 50 nmol was chosen from the results of pilot experiments showing this to be a dose providing maximal effect on late phase formalin behaviors, and from the available literature [21]. Formalin injections were performed 3 h after *i.t*. drug injections at which time drug effect was found to be maximal, in agreement with published data [21].

### Expression analysis of mouse spinal cord tissue

Microarray analyses were performed using Affymetrix GeneChip Mouse Genome 430 2.0 Array (>39,000 transcripts) using previously described methods [40]. Spinal cords were prepared from 3 experimentally naïve mice from each of the following 10 inbred strains: 129/SvIm, AKR, B10.D2-H2/oSNJ, BALB/c, C3H/He, C57BL/6, DBA/2, LP, MRL/Mp, NZW/LaC (all "J" substrains). The probe intensity data generated from all 30 arrays were read into the R software environment http://www.R-project.org directly from .CEL files using the R/affy package [41], which was also used to extract and manipulate probe level data to assess data quality and to create expression summary measures. Normalization was carried out using the robust multiarray average (RMA) method [42] to form one expression measure for each probeset on each array.

Each array has 45,101 probesets, which correspond to >39,000 transcripts. The following analyses were applied to identify probesets that were differentially expressed between strains (with a >3-fold difference). A one-way ANOVA model was applied to test whether the probeset was differentially expressed among the mouse strains. The average expression level for each probeset for each strain was calculated. Since RMA signals are on log2 scale, the fold change was defined as 2 to the power of the maximum average expression level minus the minimum expression level. Probesets with ANOVA p < 0.01 and a fold change greater than 3 were identified as genes that were significantly differentially expressed. There were 2,500 probesets that met these criteria, and these were selected for further correlational analysis with the formalin test late phase data.

### Correlational analysis of spinal cord expression and mouse behavioral data

We assessed the extent of correlation between each selected differentially expressed probeset and the phenotypes of interest using Pearson's correlation coefficient [43]. For each probeset, the RMA signal intensity (at log2 scale) was used for the correlation calculation. The probesets were then ordered by the absolute value of the correlation coefficient.

### Genomic analysis

The SNP data provided by Frazer at al. were used to identify the haplotypic structure around the *Mapk8 *gene [18]. Twelve strains were represented in this database. In addition, BALB/cBy data were available, and though we actually tested BALB/c mice, these substrains were considered genetically similar enough to include them in our haplotypic analysis. The murine *Mapk8 *gene is located on chromosome 14 from 34,191,084 to 34,260,344 bp (http://www.ensembl.org, Mouse Genome Build 37). SNP data were downloaded from http://mouse.perlegen.com/mouse/index.html, and all SNPs that were within 2,000 bp of the *Mapk8 *region were identified. There were 86 SNPs that were specific to the 8 strains within this genomic region, which included the whole *Mapk8 *gene. The haplotypic structure of this region was then visualized in Excel using a simple coloring scheme.

Halpotypic analysis was performed as outlined by Wang et al. [19] using the strain specific *Mapk8 *associated haplotype alleles. To test the degree of correlation between the strain data and the allelic identities an ANOVA based approach was used.

### Expression analysis (mRNA)

Mice were killed at specific time points by CO_2 _asphyxiation without prior exposure to formalin. Spinal cord tissue was harvested by extrusion. Lumbar spinal cord segments were dissected on a chilled surface. Dissected tissue was then quick-frozen in liquid nitrogen and stored at -80°C until use. For PCR experiments, total RNA was isolated using the RNeasy Mini Kit (Qiagen, Valencia, CA) according to the manufacturer's instructions. The purity and concentration were determined spectrophotometrically as described previously for brain and spinal cord samples [44]. In the next step cDNA was synthesized from1 μg total RNA using random hexamer priming and a First Strand cDNA Synthesis Kit (Invitrogen, Carlsbad, CA) according to the manufacturer's instructions. Quantitative PCR reactions were conducted in a volume of 4 μl using the Sybr Green I master kit (PE applied Biosystems, Foster City, CA). Using an ABI prism 7900HT system (Applied Biosystems, Foster City, California, USA), PCR was carried out using the parameters 52°C, 5 min->95°C, 10 min then (95°C, 30 s->60°C, 60 s) for 40 cycles. The sequences for the *Mapk8 *primers were (forward primer) GCCACAAATCCTCTTTCCA and (reverse primer) CACATCGGGGAACAGTTTCT. Samples were analyzed in triplicate. Melting curves were performed to document single product formation.

Quantification was accomplished according to the standard curve method as described by the PCR system manufacturer (PE Applied Biosystems) as we have used previously [45]. In order to achieve the same PCR efficiency for each analyte, serial dilution of cDNA was used to construct standards curve for *Mapk8 *and 18S RNA which was used as a control. The *r*^2 ^values for the standard curves of the genes approached 1.0, suggesting the same amplification efficiency in the PCR reactions under these conditions.

### Expression analysis (protein)

Lumbar spinal cord tissue was homogenized in SDS Lyses sample Buffer, 100 mg tissue to 1 ml sample lysis buffer mix (1.25 ml of 0.5 M Tris -HCL pH 6.8, 1 ml of glycerol, 1 ml of 20% SDS, 69 μl of β-mercaptoethanol, 6.68 ml distilled water, total 10 ml). Tissue was then vortexed followed by boiling for 8 min, then cooling on ice. The homogenate was centrifuged at 12,000 × g for 10 min at 4°C. The supernatant was collected and retained for Western blot analyses. The concentration of protein in the supernatant was measured using the DC Protein Assay kit (Bio-Rad, Hercules, CA). Equal amounts of protein (50 μg) were sized fractionated by electrophoresis on 10% acrylamide gels (Bio-Rad) and transferred onto LI-COR Odyssey Nitrocellulose Membrane (LI-COR Biosciences, Lincoln, NE). The blot was blocked for overnight in LI-COR Odyssey Blocking Buffer followed by incubation with a mixture of primary antibodies. JNK1 affinity purified rabbit polyclonal antibody was used at 1:500 dilution (Abgent, San Diego, CA) and actin mouse monoclonal antibody at 1:1000 dilution (Santa Cruz Biotechnology, Inc., Santa Cruz, CA) with incubation on a rocking platform at 4°C for 24 h. After washing, the blots were incubated 2 h at room temperature in a secondary antibody mix: IRDYE 680 donkey anti-mouse IgG (LI-COR) and IRDYE 800CW goat anti rabbit IgG (LI-COR) at 1:20,000 dilution for each. The blots were washed and placed in a LI-COR Odyssey Infrared Imaging System. Bands specific to each protein were quantified from the same gels and analyzed with LI-COR software.

### Statistical analysis

Frequency of, or total time spent licking the hindpaw as well as the time course data were analyzed by two-way ANOVA followed by post-hoc Bonferroni multiple comparison tests. A criterion α = 0.05 was employed. For expression analysis (mRNA and protein) experiments samples from n = 4 mice per strain were analyzed in triplicate. The values from three strains within each group (replicates) were compared to other strain group (High vs. low responding) by unpaired t-Test.

## List of Abbreviations

ANOVA: Analysis of variance; JNK: c-Jun N-terminal kinase; MAPK: Mitogen-activated protein (MAP) kinase; PLA2: Phospholipase A2; QTL: Quantitative trait locus; qPCR: Quantitative real time polymerase chain reaction; SEM: Standard error of the mean; SNP: Single-nucleotide polymorphism.

## Competing interests

The authors declare that they have no competing interests.

## Authors' contributions

XL performed all of the molecular biology and expression studies. PS performed the JNK inhibitor experiments and revised the manuscript. MZ identified the *Mapk8 *SNPs, determined the strain specific haplotype construction and was involved in constructing the spinal cord gene expression database. JR performed the multi-strain formalin testing experiments. GP designed and supervised the construction of the gene expression database and analysis. He also supervised the genetic analysis components of the project. JSM supervised the design of the primary multi-strain formalin testing and processing of those data. Also, he assisted in the design of the subsequent experimentation and interpretation of the resulting data. JDC coordinated the various aspects of the project and directly supervised the expression analysis and inhibitor studies.

All authors read and approved the final manuscript.
